# Morphometric and Statistical Analysis of the Palmaris Longus Muscle in Human and Non-Human Primates

**DOI:** 10.1155/2014/178906

**Published:** 2014-04-13

**Authors:** Roqueline A. G. M. F. Aversi-Ferreira, Rafael Vieira Bretas, Rafael Souto Maior, Munkhzul Davaasuren, Carlos Alberto Paraguassú-Chaves, Hisao Nishijo, Tales Alexandre Aversi-Ferreira

**Affiliations:** ^1^Department of Anatomy, Howard University College of Medicine, 520 W Street NW, Numa Adams Building, Washington, DC 20059, USA; ^2^Laboratory of Primate Anthropology, Biochemistry, Neurosciences and Behavior, Federal University of Tocantins, NS 15 Avenue, Block 109 Norte, Plano Diretor Norte, 77001-090 Palmas, TO, Brazil; ^3^Graduate School of Animal Biology, Institute of Biology, University of Brasilia, Darcy Ribeiro Campus, 70910-900 Brasília, DF, Brazil; ^4^Department of Physiology, Laboratory of Neuroscience and Behavior, University of Brasilia, Darcy Ribeiro Campus, 70910-900 Brasília, DF, Brazil; ^5^System Emotional Science, Graduate School of Medicine and Pharmaceutical Sciences, University of Toyama, Toyama 930-1494, Japan; ^6^Federal University of Rondonia, RO, Brazil

## Abstract

The palmaris longus is considered a phylogenetic degenerate metacarpophalangeal joint flexor muscle in humans, a small vestigial forearm muscle; it is the most variable muscle in humans, showing variation in position, duplication, slips and could be reverted. It is frequently studied in papers about human anatomical variations in cadavers and *in vivo*, its variation has importance in medical clinic, surgery, radiological analysis, in studies about high-performance athletes, in genetics and anthropologic studies. Most studies about palmaris longus in humans are associated to frequency or case studies, but comparative anatomy in primates and comparative morphometry were not found in scientific literature. Comparative anatomy associated to morphometry of palmaris longus could explain the degeneration observed in this muscle in two of three of the great apes. Hypothetically, the comparison of the relative length of tendons and belly could indicate the pathway of the degeneration of this muscle, that is, the degeneration could be associated to increased tendon length and decreased belly from more primitive primates to those most derivate, that is, great apes to modern humans. In conclusion, in primates, the tendon of the palmaris longus increase from Lemuriformes to modern humans, that is, from arboreal to terrestrial primates and the muscle became weaker and tending to be missing.

## 1. Introduction

The palmaris longus (PL) is considered a phylogenetic degenerate metacarpophalangeal joint flexor muscle in humans [1] and a small vestigial forearm muscle [2]; it is the most variable muscle in humans [2–4], showing variation in position, duplication, and slips [2] and could be reverted [5]. It is frequently studied in papers about human anatomical variations in cadavers and* in vivo* [6]; its variation has importance in medical clinic, for example, vascular-neural compression [7], in surgery by using its tendon for grafting or other reconstructions [2, 4, 7], in radiological analysis, in study about high-performance athletes [8], and in genetics and anthropologic studies [2].

This muscle presents significant divergences as to its frequency in different humans groups [2, 7, 9–12]; it can be absent in some individuals of the genera* Pan* and* Gorilla* [6], but it is always present in* Hylobates*,* Pongo* [9, 10], and* Sapajus* [13], and its absence has not been reported in other primates.

Most studies about PL in humans are associated with frequency or case studies, but comparative anatomy in primates and comparative morphometry were not found in scientific literature. The comparative anatomy associated to the morphometry of palmaris longus could explain the degeneration observed in this muscle in two of three of the great apes (i.e., genera* Pan* and* Gorilla*) and modern humans.

Hypothetically, the comparison of the relative length of tendons and belly could indicate the pathway of the degeneration of this muscle; that is, the degeneration could be associated with increased tendon length and decreased belly from more primitive primates to those most derivate, that is, great apes to modern humans.

Therefore, the aim this work was to compare the anatomy and to verify the relative tendon/belly length of the palmaris longus in some primates from the New World and Old World and modern humans, associating data observed with those from the literature.

## 2. Material and Methods

This work was previously approved by the Institutional Ethics Committee of the Federal University of Goiás, state of Goiás, Brazil (CoEP-UFG 81/2008), for human and nonhuman primates studied in Brazil. For other primates, the rules of animal care in the USA and Japan were followed.

### 2.1. Samples

This study evaluated 14 adult human cadavers (males), allocated at the Laboratory of Human Anatomy, Federal University of Goiás, Brazil; six exemplars of adult* Sapajus libidinosus* (5 males and 1 female) and one exemplar of adult* Callithrix* sp. (male) allocated at the Laboratory of Anthropology, Biochemistry, Neuroscience and Primates' Behavior (LABINECOP), Federal University of Tocantins, Brazil; three exemplars of adult* Macaca fuscata *(males) allocated at the Laboratory of System Emotional Science, Toyama University, Japan; and one exemplar of adult* Ateles* sp. (male), one neonate* Pongo* sp. (male), one neonate and one child* Pan* sp. (males), one adult* Callithrix* sp. (male), two* Aotus* sp., two* Lemur catta* (one male, one female), and one* Propithecus* sp. (male) allocated at the Laboratory of Evolutionary Biology, Howard University, USA.

### 2.2. Dissection, Documentation, and Measures

Human cadavers,* Sapajus* sp., and one forearm of child* Pan* sp. had been dissected for other purposes, but other specimens were dissected for purposes of this work. The muscles were photographed by digital camera and their length was measured by metallic measure tape and digital politer (Niigara Seiki model DN 150). The values of measures were standardized to specific measure of the digital politer. Three measures of each structure were made and the average was used for purpose of statistical analysis. The tendons were measured from the point no part of the belly associated to the tendon could be seen by naked eye to the palmar aponeurosis at the level of the radio's head. Some comparison data from other primates were not observed in this work and were obtained from the literature, for example,* Gorilla* sp. and* Hylobates* sp. The frequency of muscles, whenever possible, was based on scientific literature.

### 2.3. Statistical Analyses

Statistical analyses were performed using the StatPlus:mac AnalystSoft Inc./2009 software to calculate the average, standard deviation and compare the mean length values. To effect comparative anatomy, statistics was performed based on Aversi-Ferreira [14] and Aversi-Ferreira et al. [15] and data on muscle frequency, origin, and insertion from literature [10, 13] was used and compared against data of this work. To perform the statistics, simple comparative nonparametric method was used to compare two different species associated with anatomical concepts of normality and variation as nominal variables. Relative frequency (RF) was defined as

(1) $$\begin{array}{c} \text{RF}=\frac{N-nv}{N}, \end{array}$$

where *N* is the total number of specimens and *nv* is the number of individuals presenting variation of the normal pattern; therefore, RF means the structure normality in a population sample.

When more than one parameter was used, they were related to specific weighted values with respect to their degree of relevance in comparative analysis. Parameters with less variation in phylogenetic terms were assigned a higher value. Therefore, origin, insertion, innervation, and presence of muscle in terms of frequency and type of fiber arrangement were assigned weights 4, 4, 3, 2, and 1, respectively.

The weighted average of frequencies (WAF) was calculated using the RF values:

(2) PAF=RF1×P1+RF2×P2+RF3×P3+RF4×P4+RF5×P5P1+P2+P3+P4+P5,

where RF_1_ is the frequency of muscle's origin and *P*_1_ is 4; RF_2_ is the frequency of muscle's insertion and *P*_2_ is 4; RF_3_ is the frequency of innervation and *P*_3_ is 3; RF_4_ is the frequency of the presence of muscle and *P*_4_ is 2; and RF_5_ is the type of belly arrangement and the *P*_5_ is 1. The frequencies of RF to humans,* Pan* sp., and* Gorilla* sp. were obtained from Gibbs [10], who cited the absence of palmaris longus from 3.9 to 20.4%; therefore, *nv* was calculated based on the average of these numbers, that is, 12.15%, and *N* was considered to be 100%; therefore, RF to human was 0.878. The *nv* value for chimpanzee was 19 and the *N* value was 28, and RF was 0.678; for gorilla, the *nv* value was 6 and the *N* value was 19, and RF was 0.316. The type of fiber arrangement was considered to be 1 to Lemuriformes and New World primates and 0.5 to the others, because this was considered here as an intermediate character among these primates.

To consider the phylogenetic proximity between the structures studied, the difference in the relative frequency was calculated or Comparative Anatomy Index (CAI) between samples from different species:

(3) $$\begin{array}{c} \text{CAI}=\left|{\text{PAF}}_{i}-{\text{PAF}}_{ii}\right|, \end{array}$$

where indices *i* and *ii* represent samples 1 and 2.

From the previous equations, it follows that CAI value close to zero represents greater similarity between samples, whereas CAI closer to 1.0 implies higher divergence between samples. Therefore, the greater the numerical distance between values, the greater the divergence in relation to the palmaris longus muscle between species.

## 3. Results

**Figure 1:**
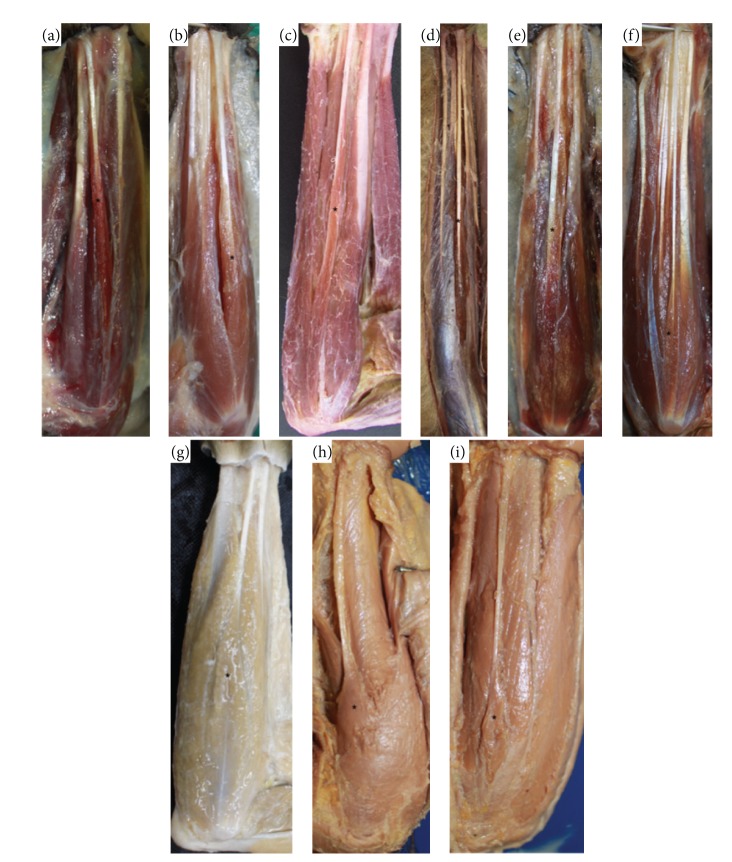
Photos of the forearms of (a)* Propithecus* sp. (0.45x, left); (b)* Lemur catta* (0.52x, left); (c)* Sapajus libidinosus* (0.34x, right); (d)* Ateles* sp. (0.1x, right); (e)* Callithrix* sp. (0.3x, right); (f)* Aotus* sp. (0.8x, right); (g)* Macaca fuscata* (0.34x, right); (h)* Pongo* sp. (0.79x, left); (i)* Pan* sp. (0.23x, left). From (a) to (f), muscles are pennate and from (g) to (i) are fusiform. ∗ indicates the palmaris longus.

In all primates studied here, the PL tendon, originated from medial humeral epicondyle and inserted into the palmar fascia, was innervated by the median nerve. A close relationship between the palmaris longus tendon and the fascia of the forearm was observed, which is similar to other flexor muscles of the forearm. The belly of palmaris longus was easily distinguished from the other muscles of the forearm in all studied species, except for the only exemplar of* Ateles* sp., in which the flexor muscles formed one group of bellies from the elbow with a separation of tendons close to the wrist (Figure 1).

**Figure 2:**
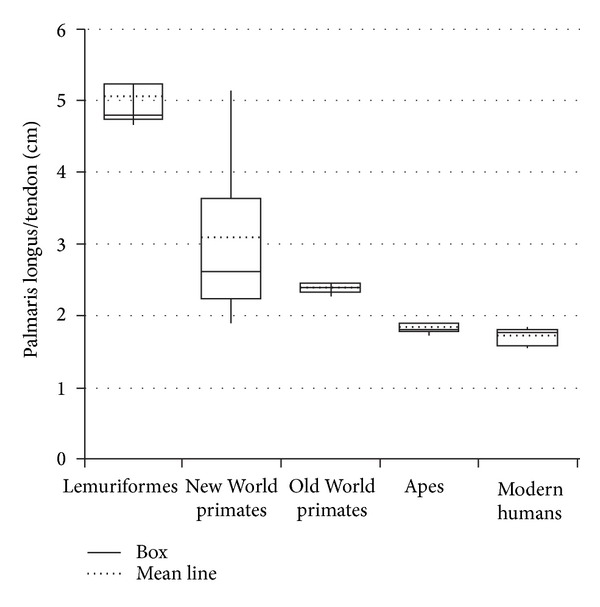
Graph showing the mean line relative to the palmaris longus/tendon length of primate groups.

Regarding the type of fiber arrangement in the belly, the palmaris longus tendon presented an aspect similar to pennate in all Lemuriformes and New World primates (Figure 1), but fusiform to* Macaca fuscata* (unique exemplar of Old World primates) and to apes (*Pongo* sp. and* Pan* sp.) and modern humans. The average palmaris longus/tendon length showed significant differences between species studied in this work (Table 1, Figure 2). Shorter tendons were observed in Lemuriformes and longer in modern humans (*P* < 0.05, Figure 2).

Significant differences were observed between averages (*P* < 0.05) calculated for all groups, that is, Lemuriformes and other groups, New World primates and other groups, and Old World primates (*Macaca fuscata*) and other groups, and for apes and modern humans (Table 1, Figure 2). Nevertheless, to* Aotus* sp., specifically, in comparison to* Macaca fuscata* and apes, differences were not observed.

The CAI indicates that Lemuriformes and New World primates share similar characters to PL (CAI = 0) while these features are more derived in* Gorilla* sp. (CAI = 0.133) (Table 2).

## 4. Discussion

The large variations in the prevalence of the PL among modern humans may be indicative that this muscle is degenerating [1], and its small belly may suggest it is a vestigial muscle [2].

Although there are several studies investigating the frequency of PL in modern humans, only a few recent studies have compared PL frequency in nonhuman primates [9, 10, 16–18]. From such studies, it was found, for example, that the frequency of PL in* Gorilla* sp. is much lower than in humans and chimpanzees, even though humans and chimpanzees are the only species in which absence of PL has been reported. The PL is found in* Gorilla* sp. from 15% to 63% according to some authors [10, 16, 17, 19, 20] and could be considered absent in gorillas according to [18].

In this sense, considering only the frequency of this muscle in primates, PL would be more degenerative in* Gorilla* sp. Also, if degeneration is considered a derived characteristic of PL, it is more derived in* Gorilla* sp. than in other primates and modern humans. Indeed, it is reasonable to suggest that PL is a degenerative muscle because, according to Meglioli [21], its agenesis is associated with a recessive gene.

Notwithstanding, when parameters such as origin, insertion, innervation, frequency and type of belly fibers are compared together and using specific weight, (i.e., when CAI is applied; for review, see Aversi-Ferreira [14] and Aversi-Ferreira et al. [15]) Gorilla sp. shows the greatest numerical distance from the primitive character considered in Propithecus sp., followed by Pan sp., modern humans, Pongo sp. and Macaca fuscata in decreasing order.

In fact, the different variation factor observed in the CAI calculation can be explained by the low frequency in the number of specimens and species and by the different types of muscle fibers of the belly muscle obtained from the literature and from the present study. To Lemuriformes and New World primates, CAI indicates no quantitative difference; that is, the calculated value is zero; but to* Macaca fuscata* and* Pongo* sp., the only difference is type of fiber arrangement.

In order to obtain more objective parameters, the relative length of the tendon was studied, that is, the length of the PL muscle divided by the length of the palmaris longus tendon. Its measure indicates, indirectly, the muscle relative strength because smaller belly indicates less sarcomeres acting together to generate contraction [22]. Therefore, a longer tendon indicates less muscular force. According to the data obtained here, this relation (PL muscle divided by the length of the palmaris longus tendon) decreases from Lemuriformes to modern humans.

Interestingly, the comparison between means of the relative measures of PL among these groups of primates (*P* < 0.05) showed significant differences between one group and all other groups. One discrepancy was observed in the measures obtained from* Aotus* sp., a New World primate, in which the average measures were similar to* Macaca fuscata* and apes, but it was not possible to associate it with any aspect considered in this work. Putatively, a weak PL is observed in modern humans and apes and strong in Lemuriformes. Therefore, it is possible to hypothesize that the tendon length decreases from arboreal to terrestrial primates. In line with this, Ankel-simons [23] reports that great apes move in a quadrupedal Knuckle-walking manner; the* Macaca* genus is terrestrial quadruped; all New World primates are highly arboreal, and all Lemuriformes, except for* Lemur catta*, spend around 1/3 of the time on the ground.

Specifically to modern humans, the average measure of the PL tendon obtained here, that is, 15.06 (±1.75), was different from those reported for other Brazilians cadavers [12] (11.99 ± 1.52). Apparently, these differences are due to criteria for length measurements of the palmaris longus tendon in both studies.

On the other hand, different fiber arrangement of the belly of PL in Lemuriformes and New World primates compared to Old World primates, apes, and modern humans was observed. The tendon begins in the belly, which involves the tendon laterally up to approximately the wrist in Lemuriformes and New World primates, characterizing a pennate muscle; whereas from* Macaca fuscata* to modern humans, the tendon begins after the end of the belly, observed via naked eye, characterizing a fusiform muscle.

This characteristic affects the muscular strength, the fusiform muscle generates a more direct contraction, but to modern humans, apes, and* Macaca fuscata*, the bellies are shorter; in a pennate muscle, however, the physiological cross-sectional area is considerably larger than its anatomical cross-sectional area. Therefore the pennate muscle, ceteris paribus, generates more force [24]. In addition, they are longer in Lemuriformes and New World primate than in other primates.

The number of animals per species and the number of species used here allowed inferring the following conclusions. First, the more derived characteristics of PL are found in apes, especially in* Gorilla* sp. relative to evolutionary aspects measured by CAI, but a shorter tendon is verified in modern humans. Second, the path to the degeneration of the PL seems to follow a decreased tendon associated with modification in the type of belly fibers from pennate to fusiform; therefore, in primates with more derived thoracic limbs, the weaker PL.

To our knowledge this is the first study to present an evolutionary anatomical comparison of the PL. Further studies could indicate more accurately the evolutionary pathway followed by PL from primitive to derived primates associated with aspects such as the muscular force difference between primates and verification of anatomical aspects in more species and consider intra- and interspecific variations

## 5. Conclusions

Apparently, in primates, the PL tendon relative size seems to increase from ancestors genera to more derived ones. This suggests that this muscle is weaker in terrestrial primates when compared to arboreal primates. The PL tendon seems to be more derived in* Gorilla* sp., considering the factors analyzed here, but the tendon is longer in modern humans.

## Figures and Tables

**Table 1:** Measures of the palmaris longus, palmaris longus tendon, and palmaris longus/tendon relationship for species of primates and primate groups.

Specimens (*n*)	Total	Tendon	Total/tendon	Primate groups
Modern humans (28)	25.51 (±1.69)	15.06 (±1.75)	1.71 (±0.13)	Modern humans^†, Δ, ∗, +^
*Pan *sp. (3)	8.83 (±1.62)	4.95 (±0.91)	1.78 (±0.04)	Apes^†, Δ, ∗, +^
*Pongo *sp. (2)	8.30 (±0.10)	4.40 (±0.30)	1.89 (±0.15)	
*Macaca fuscata *(6)	17.30 (±0.43)	7.30 (±0.5)	2.37 (±0.12)	Old World primates^†, Δ, ∗^
*Aotus *sp. (4)	6.8 (±0.47)	3.2 (±0.50)	2.09 (±0.23)	New World primates^†, Δ^
*Callithrix *sp. (3)	4.35 (±0.19)	1.72 (±0.23)	2.53 (±0.08)	
*Ateles * sp. (1)	25.3	10.0	2.53	
*Sapajus libidinosus *(11)	10.71 (±1.50)	2.99 (±0.51)	3.81 (±1.07)	
*Lemur catta* (2)	8.6 (±0.50)	1.9 (±0.0)	4.53 (±0.27)	Lemuriformes^†^
*Propithecus *sp. (2)	11.25 (±0.05)	2.2 (±0.20)	5.16 (±0.49)	

^†^Significant difference among Lemuriformes and other groups.

^Δ^Significant difference among New World primates and other groups.

*Significant difference among Old World primates and other groups.

^+^Significant difference between apes and modern humans.

**Table 2:** Statistics of the comparative anatomy of palmaris longus.

Taxon	PAF	$\text{PAF}=\frac{\text{R}{\text{F}}_{\text{1}}\times P_{1}+\text{R}{\text{F}}_{\text{2}}\times P_{2}+\text{R}{\text{F}}_{\text{3}}\times P_{3}+\text{R}{\text{F}}_{\text{4}}\times P_{4}+\text{R}{\text{F}}_{\text{5}}\times P_{5}}{P_{1}+P_{2}+P_{3}+P_{4}+P_{5}}$	CAI = |PAF_*i*_ − PAF_*ii*_|
*Propithecus *sp.	1	$\text{PAF}=\frac{4\times 1+4\times 1+3\times 1+2\times 1+1\times 1}{14}$	Reference (most primitive characters)
*Lemur catta *	1	$\text{PAF}=\frac{4\times 1+4\times 1+3\times 1+2\times 1+1\times 1}{14}$	0
*Callithrix *sp.	1	$\text{PAF}=\frac{4\times 1+4\times 1+3\times 1+2\times 1+1\times 1}{14}$	0
*Sapajus *sp*. *	1	$\text{PAF}=\frac{4\times 1+4\times 1+3\times 1+2\times 1+1\times 1}{14}$	0
*Ateles *sp.	1	$\text{PAF}=\frac{4\times 1+4\times 1+3\times 1+2\times 1+1\times 1}{14}$	0
*Macaca fuscata *	0.964	$\text{PAF}=\frac{4\times 1+4\times 1+3\times 1+2\times 1+1\times 0.5}{14}$	0.036
*Pongo *sp.	0.964	$\text{PAF}=\frac{4\times 1+4\times 1+3\times 1+2\times 1+1\times 0.5}{14}$	0.036
Modern humans	0.947	$\text{PAF}=\frac{4\times 1+4\times 1+3\times 1+2\times 0.878+1\times 0.5}{14}$	0.053
*Pan *sp.	0.918	$\text{PAF}=\frac{4\times 1+4\times 1+3\times 1+2\times 0.678+1\times 0.5}{14}$	0.082
*Gorilla *sp.	0.867	$\text{PAF}=\frac{4\times 1+4\times 1+3\times 1+2\times 0.316+1\times 0.5}{14}$	0.133

PAF is the weighted average of frequencies; CAI is Comparative Anatomy Index.
